# Lyssavirus Antibody Detection in Cave-Dwelling Bats on Cat Ba Island, Vietnam: Implications for Zoonotic Surveillance

**DOI:** 10.3390/vetsci12070654

**Published:** 2025-07-11

**Authors:** Heliana Dundarova, Tsenka Chassovnikarova, Nadezhda Todorova, Michaela Beltcheva, Iliana Aleksieva, Nguyen Thanh Luong, Vu Dinh Thong

**Affiliations:** 1 Institute of Biodiversity and Ecosystem Research, Bulgarian Academy of Sciences, 1 Tsar Osvoboditel, 1000 Sofia, Bulgaria; 2 Department of Zoology, Faculty of Biology, University of Plovdiv “Paisii Hilendarski”, 24 Tzar Asen Str., 4000 Plovdiv, Bulgaria; 3 Institute of Biology, Vietnam Academy of Science and Technology (VAST), 18 Hoang Quoc Viet Road, Nghia Do Ward, Cau Giay District, Hanoi 10072, Vietnam; 4 Faculty of Biology, Graduate University of Science and Technology, Vietnam Academy of Science and Technology (VAST), 18 Hoang Quoc Viet Road, Nghia Do Ward, Cau Giay District, Hanoi 10072, Vietnam

**Keywords:** Lyssavirus prevalence, Chiroptera, underground sites

## Abstract

Southeast Asia is a biodiversity hotspot for bats that can carry lyssaviruses, causing zoonotic diseases. This study detects and quantifies IgG antibodies against *Lyssavirus* glycoproteins in cave-dwelling bat populations on Cat Ba Island, northern Vietnam, to determine their past exposure history and the prevalence of immune responses. Samples were collected from five caves, encompassing three families and five key species (*Hipposideros armiger*, *H. alongensis*, *H. poutensis*, *Taphozous melanopogon*, and *Myotis pilosus*). Using ELISA with the Platelia™ Rabies II kit,(Bio-Rad Laboratories, Marnes-la-Coquette, France) 29.0% (18/62) of the bats tested positive, indicating prior exposure. The detection rate was slightly higher in females (35.7%) than in males (30.4%). *Lyssavirus*-specific antibodies were detected in four species, with the highest levels found in *M. pilosus*, followed by *H. alongensis*, *H. armiger*, and *H. poutensis;* no positives were found in *T. melanopogon* samples. One bat exhibited high seroconversion value (>4 EU/mL). The findings provide serological evidence of widespread lyssaviruses exposure in asymptomatic bats on Cat Ba Island, confirming their role as reservoirs that elicit an immune response without exhibiting rabies symptoms. This highlights the role of caves in facilitating close contact among bats, which may increase viral transmission, highlighting the need for continued surveillance in these unique roosting environments.

## Simple Summary

Vietnam is a significant hotspot for global biodiversity, particularly in its karst regions. The island of Cat Ba, located in the northern part of the country, features extensive cave systems that are home to diverse bat communities. We detected and quantified IgG anti-rabies viral glycoprotein in four out of the five studied cave-dwelling bat species on Cat Ba Island. These bat populations harbor lyssavirus loads, with potential implications for human and animal health.

## 1. Introduction

Bats (Chiroptera) represent the second largest and most widely distributed order within Mammalia, accounting for over 20% of existing mammalian species [1]. As the only mammals capable of active flight, bats occupy a broad range of ecological niches and are globally distributed. Bat species richness is remarkably high in the Asian tropics and subtropics, exhibiting substantial taxonomic and ecological diversity [2,3]. The migratory behavior, broad geographic distribution, and longevity of bats contribute to their susceptibility to serving as reservoirs for a wide range of bacterial, parasitic, and viral pathogens. The remarkable diversity of viruses found across different bat families supports the hypothesis of a long-term co-evolutionary relationship with these pathogens [4], resulting in strong innate immune mechanisms that allow them to tolerate or suppress severe viral infections. Although rodents exhibit greater species richness overall, bats are known to host more zoonotic viruses per species [5]. Notably, approximately 85% of the viral sequences found in bats are classified as RNA viruses, which are also responsible for most emerging infectious diseases [6].

Southeast Asia is recognized as a biodiversity hotspot for bats, with over 200 species documented in the region [7,8,9]. The area is also critical for emerging and re-emerging infectious diseases, as bats are frequently identified as natural reservoirs for numerous zoonotic pathogens [10]. Within this region, Cat Ba Island in Northern Vietnam exhibits substantial ecological diversity, with extensive karst ecosystems that serve as optimal roosting habitats for a diverse array of bat species [11] and support diverse communities of cave-dwelling bats [12]. These bat populations may harbor significant pathogen loads, which could impact human and animal health. For instance, the prevalence of rabies in Vietnam remains an ongoing concern, with approximately 100 human fatalities reported annually, primarily attributable to transmission by domestic dogs [13]. This combination of high bat biodiversity, accessible natural habitats, and a documented history of emerging infectious diseases underscores the region’s potential for zoonotic spillover events, including the transmission of lyssaviruses to humans and other animals. Despite the inherent risks, Asia, including Vietnam, remains the least surveyed continent concerning bat-associated lyssaviruses, highlighting a critical gap in surveillance and understanding of their distribution and diversity [8,14].

Bats are widely recognized as the primary reservoir hosts for various emerging viruses [15,16]. A combination of physiological, immunological, and life history factors, many of which are directly related to flight, helps classify them as “special” disease reservoirs [17]. These include synchronized birthing cycles, large communal populations formed by gregarious species, the use of torpor and hibernation in some bats, daily increases in body temperature during flight (known as the “flight fever hypothesis”), and unique immune system features [15,16,18,19].

According to other authors, bats often contract viruses, recover, and develop immunity by producing high levels of Abs, typically without becoming reservoirs for the viruses [20]. They have a balanced immune response that is slow but highly efficient. This makes them seropositive and immune to viruses, but not to the point of becoming contagious [21]. Higher expression of viral glycoprotein lyssaviruses has been linked to increased apoptosis and a more robust immune response, potentially leading to viral attenuation or milder disease [22]. This mechanism clarifies how bats can harbor lyssaviruses asymptomatically, as their immune systems may effectively control the infection [21]. Constitutive high interferon (IFN) expression in bats also plays a key role in maintaining robust innate immune responses, serving as the first line of defense against pathogens and leads to the ability of bats to coexist with viruses in the absence of disease [23]. This pre-existing antiviral state limits viral replication, boosts natural killer cell activity, and enhances antigen presentation, thereby reducing pathogen spread and preparing the adaptive immune system for a more focused response [24].

The ability of bats to influence the evolution, distribution, and ecology of lyssaviruses is notable [14]. The lyssaviruses, members of the Rhabdoviridae family, are the etiological agents of rabies, a highly lethal zoonotic disease characterized by pronounced neurotropism [25] that affects all mammals, leading to acute progressive encephalomyelitis [26]. The prototype species, rabies lyssavirus (RABV), is the most prevalent of all lyssaviruses and poses a significant global public health concern [26]. Rabies has a disproportionate burden of human deaths reported in Asia, highlighting the continued need to clarify the epidemiology of the virus in this region [27]. This invariably fatal zoonotic disease is primarily transmitted through bites or scratches from infected animals or contact with their saliva, mucous membranes [28]. Globally, rabies accounts for an estimated 59,000 human deaths annually, with over 95% occurring in Africa and Asia, predominantly linked to canine transmission [29]. Historically, control efforts have primarily targeted the classical rabies transmitted by domestic animals, especially dogs. However, bats are now recognized as natural reservoirs of lyssaviruses, including RABV [29].

The genus *Lyssavirus* comprises enveloped, single-stranded RNA viruses. To date, two species of bat lyssaviruses, Taiwan Bat Lyssavirus 1 (TWBLV1) and Taiwan Bat Lyssavirus 2 (TWBLV2), have been identified in Southeast Asia [30,31]. These viruses belong to phylogroup I of the genus *Lyssavirus*, which is widely distributed in the Eurasian region [32]. The prototypical virus within the genus *Lyssavirus* is rabies virus (RABV) [33], which exhibits a worldwide distribution and primarily infects domestic dogs and wild carnivores [34,35]. However, lyssaviruses belonging to phylogroup I have been detected in various bat species, including insectivorous, frugivorous, and nectarivorous bats, demonstrating a broad host range and ecological adaptability [36].

Enzyme-linked immunosorbent assay (ELISA) is a widely accepted quantitative technique for detecting viral exposure, including in bats. The identification of specific Abs against a pathogen in animals is generally more reliable and widely used [37,38] than the direct identification of the virus itself, which can fluctuate in circulation and migration to target organs over time. This technique is particularly well-suited for the detection and quantification of *Lyssavirus*-specific Abs in bats due to its versatility, high sensitivity, minimal infrastructure requirements, and favorable safety profile. Additionally, it offers rapid turnaround times and is amenable to automation for high-throughput population screening [39]. The PLATELIA™ RABIES II kit (Bio-Rad Laboratories, Marnes-la-Coquette, France) demonstrates high specificity. It serves as a valuable tool for the in vitro detection of *Lyssavirus* anti-glycoprotein Abs in a range of carnivorous animals (e.g., dogs, cats, foxes), which are known reservoirs of the lyssaviruses [40]. Given the phylogenetic diversity among these species, the secondary Abs included in the kit may exhibit broad cross-species reactivity, particularly through recognition of conserved regions of the IgG molecule that act as “universal” Abs determinants; therefore, they may also have probable application in bat Abs repertoire studies. An additional advantage is that the kit detects anti-*Lyssavirus* glycoprotein-specific Abs, which are predominantly targeted by the host immune system; thus, it provides a reliable indicator of prior viral exposure and immunological response.

The effects of lyssaviruses infections in bats and their patterns of circulation within bat populations remain incompletely understood. Consequently, further research is needed to enhance the understanding, monitoring, and prevention of rabies cases associated with bats. The potential emergence of novel variants of lyssaviruses from bat reservoirs highlights the critical need for comprehensive surveillance, especially in regions with high bat diversity and increasing human–wildlife interactions. In this context, the present study aimed to detect and quantify immunoglobulin G (IgG) Abs against the glycoproteins of lyssaviruses in various cave-dwelling bat populations on Cat Ba Island in northern Vietnam, to ascertain their past exposure history and the prevalence of immune responses.

This is the first investigation of its kind conducted in this region. Given the island’s unique ecological context and the recognized role of bats as reservoirs for lyssaviruses, it is hypothesized that the detection of *Lyssavirus*-specific Abs in cave-dwelling bats on Cat Ba Island may provide valuable insights into the potential epidemiological implications of viral exposure for both human and animal populations in the region.

## 2. Materials and Methods

### 2.1. Study Sites

**Figure 1:**
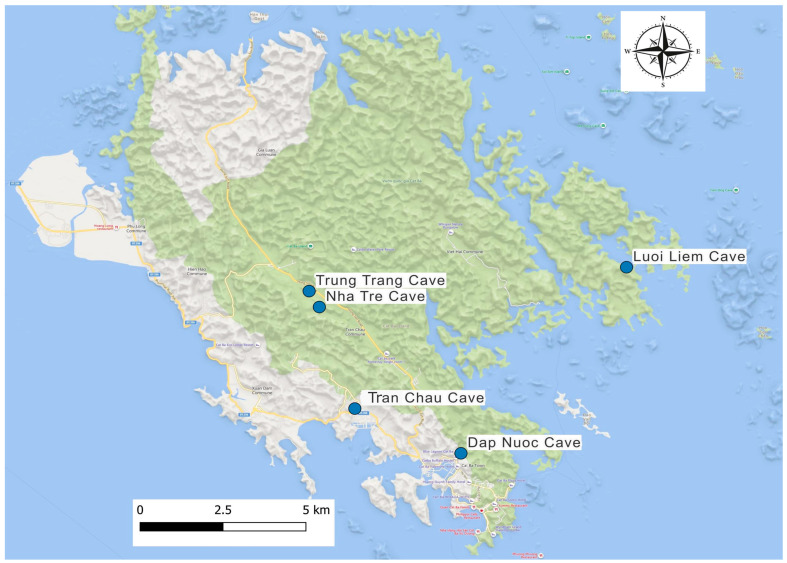
Sampling sites on Cat Ba Island, Vietnam.

The study was conducted on Cat Ba Island, located in northern Vietnam, characterized by its karst topography and numerous caves. Sampling sites included Nha Tre Cave, Luoi Liem Cave, Trung Trang Cave, Tran Chau Cave, and Dap Nuoc Cave, which were strategically selected based on specific ecological and epidemiological criteria crucial for assessing pathogen presence and zoonotic risk (Figure 1). Ecologically, these caves serve as optimal roosting habitats for various bat species, each playing a different role in pathogen ecology. Epidemiologically, increased human–bat interactions in these caves significantly raise the risk of pathogen spillover transmission.

### 2.2. Sample Collection

Bats were captured using mist nets and harp traps. A total of 62 individuals were sampled across five caves: Nha Tre Cave (n = 17), Luoi Liem Cave (n = 15), Trung Trang Cave (n = 14), Tran Chau Cave (n = 13), and Dap Nuoc Cave (n = 3).

The captured bats were identified at the species level and classified into three families: Emballonuridae, Hipposideridae, and Vespertilionidae. The species recorded were *Hipposideros armiger* (n = 18), *H. alongensis* (n = 18), *H. poutensis* (n = 18), *Taphozous melanopogon* (n = 2), and *Myotis pilosus* (n = 6).

Blood samples (25 µL each) were collected from the uropygial vein using sterile pipette tips. Samples were immediately transferred to heparinized microtubes for anticoagulation. A drop of tissue adhesive (Surgibond, Surgical Medical Instruments AG, St. Vith, Belgium) was applied to the puncture site to promote hemostasis and reduce the risk of continued bleeding. This sampling method is considered minimally invasive. All sampled bats were administered oral vitamin supplements before release. To minimize stress and potential harm to the bats during sampling, each individual was handled for no longer than 30 min. Only a minimal amount of blood was collected. After the procedure, each bat was orally administered Duphalyte—a veterinary solution containing dextrose for immediate energy and electrolytes to replace lost fluids and speed recovery. All bats successfully flew away after handling, and no dead bats were seen in the roosting sites during later habitat checks.

Capture and sampling procedures were conducted under research permits authorized by the Hai Phong City People’s Committee, Vietnam (License № 824/UBND-MT, issued 12 April 2024). All activities involving the capture and handling of animals, whether in the field or laboratory, were performed following ethical guidelines established by the Animal Ethics Committee of the Institute of Biodiversity and Ecosystem Research, Bulgarian Academy of Science, and were fully compliant with Directive 2010/63/EU of the European Parliament and of the Council on the protection of animals used for scientific purposes.

### 2.3. Detection of Lyssavirus-Specific Antibodies Using the PLATELIA RABIES II Kit

*Lyssavirus*-specific Abs were detected using the PLATELIA RABIES II kit (Bio-Rad Laboratories, Marnes-la-Coquette, France) according to the manufacturer’s instructions. For each sample, the presence and quantification of Abs targeting the *Lyssavirus glycoprotein* were assessed after a 1:100 serum dilution. The initial dilution was made in R6 reagent, a red-colored TRIS-EDTA buffer (included in the PLATELIA™ RABIES II kit, Bio-Rad Laboratories, Hercules, CA, USA). The ELISA procedural steps were conducted according to the manufacturer’s specifications, and absorbance was measured at 450 nm using a PR 4100 Absorbance Microplate Reader (Bio-Rad Laboratories, Hercules, CA, USA).

The PLATELIA™ RABIES II (Bio-Rad Laboratories, Marnes-la-Coquette, France) kit utilizes a glycoprotein derived from inactivated and purified virus membranes. The plate is coated with native G protein from a reference strain, the Pasteur Virus (PV) strain, which is widely recognized and used in diagnostics of lyssaviruses and vaccine production [40]. The kit’s controls are being calibrated against OIE standards (for veterinary use), reflecting its relevance to global benchmarks.

According to the PLATELIA™ RABIES II kit (Bio-Rad Laboratories, Marnes-la-Coquette, France) manual, the interpretation thresholds are (a) sufficient seroconversion/protective threshold for vaccinated domestic animals (dogs, cats, foxes) (≥0.5 EU/mL), (b) lower analytical limit of detection/quantitation (≥0.125 EU/mL), indicating Abs presence (above background noise), and (c) <0.125 EU/mL, which is generally considered negative or undetectable.

Since the kit was designed to assess vaccination levels in domestic animals, we employed broader criteria to interpret results in bats, considering the specific context of immune responses in this new host, which may exhibit different immune kinetics or baseline antibody levels. Lower, yet detectable, antibody levels may still indicate a cleared infection, transient exposure, or a low-level persistent immune response. The kit has been validated for detecting Abs in various species, including domestic animals and wildlife, with a lower detection limit of 0.125 EU/mL [41]. The manufacturer specifies a reported range of 0.125 to 0.5 EU/mL as indicative of antibody presence. A similar range, interpreted as indicating low seroconversion rates, was used in previous studies focused on wildlife populations of carnivores and bats [42,43,44,45,46,47]. Therefore, we considered any detectable level ≥0.125 EU/mL to exceed the negative threshold and indicate prior exposure.

To statistically validate the pre-selected, biologically informed cutoff for antibody levels within our bat cohort, we performed a receiver operating characteristic (ROC) curve analysis. For this analysis, we defined individuals with antibody concentrations below 0.125 EU/mL as a “negative” group, serving as the presumptive unexposed baseline within our sample. While these did not constitute the external controls, this method enabled a data-driven determination of the optimal threshold for our specific context, recognizing the inherent logistical, ethical, and practical difficulties in establishing true baseline controls for natural exposure in wild populations.

### 2.4. Statistical Analysis

Statistical analyses were conducted utilizing Prism software, version 9.5.1 (GraphPad Software, San Diego, CA, USA). Descriptive statistics, including means and standard deviations, were computed. A receiver operating characteristic (ROC) curve analysis was performed, and both the Area Under the Curve (AUC) and the Youden index were calculated to validate a pre-selected, biologically informed cutoff for Abs levels in bats, even in the absence of external controls. This methodology facilitates the assessment of diagnostic accuracy, which is advantageous in the study of novel lyssaviruses hosts, such as bats. To calculate the percentage of positive samples and their 95% confidence intervals, an OpenEpi calculator for epidemiologic statistics in public health was used (https://www.openepi.com/Proportion/Proportion.htm, accessed on 29 April 2025).

## 3. Results

### 3.1. ROC Curve Analysis for Diagnostic Validation

**Figure 2:**
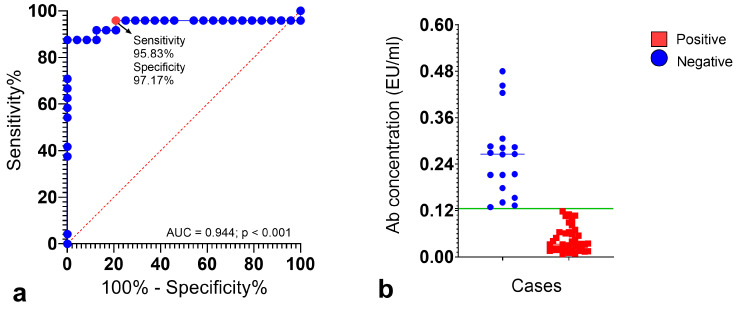
Receiver operating characteristic (ROC) curve, derived directly from the studied bat population via PLATELIA™ RABIES II kit, (Bio-Rad Laboratories, Marnes-la-Coquette, France); (**a**) ROC curve showing the accuracy value, reflected by the area under the curve (AUC); (**b**) scatter plot showing the discrimination power of the chosen threshold value (green line) and its diagnostic sensitivity and specificity.

A receiver operating characteristic (ROC) curve was plotted to evaluate the diagnostic performance of the PLATELIA^TM^ RABIES II kit (Bio-Rad Laboratories, Marnes-la-Coquette, France) in wild bat populations. It enabled the provision of a statistically validated framework to assess antibody levels’ cutoff points and optimize the diagnostic utility. Figure 2a illustrates that the Area Under the Curve (AUC), a robust index of the assay’s discriminating ability, was 0.944 (*p* < 0.001), with a 95% confidence interval (CI) ranging from 0.862 to 1.000. This high AUC value indicates the assay’s strong discriminatory power in the context of the studied bat samples. The corresponding Youden index of 0.93 further confirms the test’s discriminatory ability at this operating point. This statistically derived point approaches the ideal top-left corner of the ROC curve, indicating a robust balance between correctly identifying both likely exposed and likely unexposed cases. This finding provides internal validation for our initial biologically informed decision to set a threshold of ≥0.125 EU/mL, demonstrating that even at this lower level, the assay maintains high discriminatory accuracy for detecting *Lyssavirus* Abs in our bat samples (Figure 2b). The ROC analysis provides statistical evidence supporting the reliability of using Ab levels at or above this range to distinguish individuals with evidence of prior lyssaviruses exposure from those without, within our study population.

### 3.2. Serological Survey of Cat Ba Cave-Dwelling Bats

**Figure 3:**
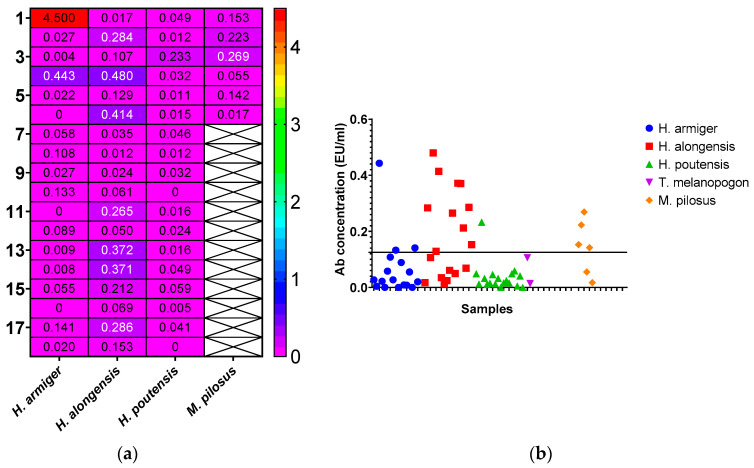
(**a**) Heatmap showing concentrations (EU/mL) of *Lyssavirus*-anti-glycoprotein antibodies among cave-dwelling bats on Cat Ba Island. Legend: Each row shows a different bat sample with its Lyssavirus anti-glycoprotein antibody levels, while each column corresponds to a bat species; pink indicates the lowest concentrations, red the highest, and violet signifies values above 0.125 (EU/mL) that surpass the negative threshold and are considered Lyssavirus-positive; black/white font color indicates lack of samples; (**b**) The concentration distribution of *Lyssavirus*-anti-glycoprotein antibodies (EU/mL) in cave-dwelling bats on Cat BA Island, North Vietnam (2024). The black bar signifies antibody threshold levels exceeding 0.125 EU/mL, classifying the corresponding individuals as lyssavirus-positive.

Our findings indicate that bats on Cat Ba Island had detectable titers of *Lyssavirus*-specific Abs, demonstrating an immune response against lyssaviruses. Four of the five bat species studied (*H. armiger*, *H. alongensis*, *H. poutensis*, and *M. pilosus*) exhibited detectable levels of *Lyssavirus*-specific Abs in their peripheral blood (Figure 3a), exceeding the 0.125 EU/mL seropositivity threshold (Figure 3b). Only one sample (Figure 3a) exhibited a high seroconversion value (>4 EU/mL).

Overall, 18 of the 62 animals (29.0%; 95% confidence interval (CI): 19.2–41.3%) showed detectable levels of Abs against lyssaviruses (Table 1). The positive rate among females was 35.7% (95% CI: 16.4–61.2%), while it was 30.4% (95% CI: 19.0–46.84%) in males. *Lyssavirus*-specific Abs were detected in four of the five bat species identified: *H. armiger* (n = 4 positive individuals), *H. alongensis* (n = 10), *H. poutensis* (n = 1), and *M. pilosus* (n = 4). These positive individuals accounted for 6.7%, 16.7%, 1.7%, and 6.7% of all bats tested by ELISA, respectively (Table 1). None of the samples from *T. melanopogon* tested positive (Table 1). The highest total positive percentage was observed in *M. pilosus* individuals, followed by *H. alongensis*, *H. armiger*, and *H. poutensis*.

### 3.3. Sex-Related Intraspecies Differences

**Figure 4:**
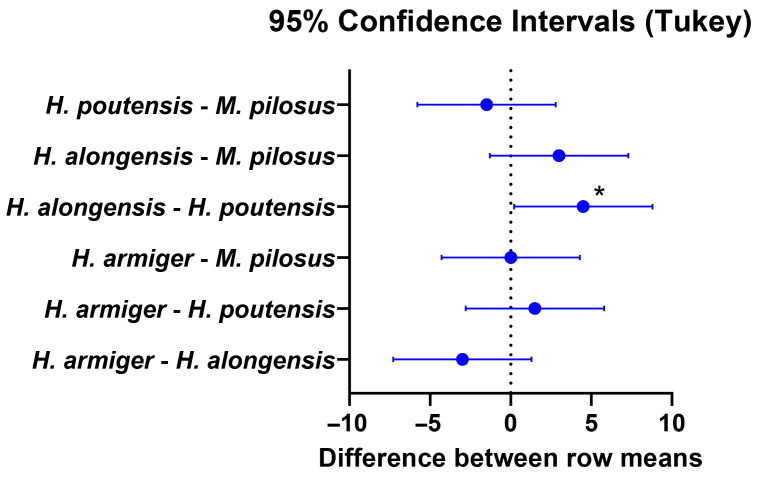
Means and 95% confidence intervals found in bats positive for *Lyssavirus*-specific anti-glycoprotein antibodies revealed by two-way ANOVA with Tukey post hoc test. The asterisk indicates statistical significance at *p* < 0.05.

A two-way ANOVA with Tukey’s multiple comparisons test was conducted to evaluate sex-related statistical significance among *Lyssavirus*-positive individuals (Figure 4). A significant difference was identified between the sexes in *H. alongensis* and *H. poutensis* (*p* < 0.05).

## 4. Discussion

### 4.1. Serological Evidence of Lyssavirus Circulation

Rabies is one of the oldest zoonotic viral diseases, affecting both humans and animals. Bats serve as the ancestral reservoir for lyssaviruses, including RABV, sustaining numerous distinct lyssaviruses lineages worldwide, with at least 12 of the 14 recognized species. Due to the diversity of lyssaviruses in bats, it is believed that their ancestors have had a significant influence on the evolutionary history of these species [48]. These ancestral viruses likely adapted to bats in the Old World, possibly in Africa or Eurasia, regions currently home to the most extraordinary lyssaviruses diversity. The dynamics of rabies morbidity in bats differ from those in terrestrial animals in terms of the distribution and pathogenicity of lyssaviruses, and knowledge in this area remains incomplete.

This research demonstrates the detection and quantification of IgG anti-*Lyssavirus* glycoprotein in four out of the five cave-dwelling bats studied on Cat Ba Island, Northern Vietnam. Our study employed a cutoff value of 0.125 EU/mL to define positive anti-*Lyssavirus* Abs samples, which is lower than the manufacturer’s recommended threshold for general use. This decision was based on several critical considerations relevant to serological surveillance in bat populations. The PLATELIA RABIES II kit (Bio-Rad Laboratories, Marnes-la-Coquette, France) is validated for diverse species. It has a lower detection limit of 0.125 EU/mL [49]. The existing literature suggests that bats, such as *Desmodus rotundus* and *Eptesicus fuscus*, can develop detectable neutralizing Abs levels against RABV, even at low thresholds (e.g., 0.1–0.5 IU/mL), as demonstrated by methods like the Rapid Fluorescent Focus Inhibition Test (RFFIT) [44,45]. Furthermore, the use of the 0.125 EU/mL threshold in our ELISA is supported by previous serological testing on Palearctic bats by [46], who employed the same ‘low seroconversion’ threshold of 0.123 EU/mL, derived from a calibration curve. This consistency with other bat studies strengthens the validity of our approach for detecting a broader range of Ab responses in this specific host group. These lower Abs levels could indicate past, sub-clinical infections or weak immune responses, which are nevertheless biologically relevant in the context of epidemiology of lyssaviruses in wildlife. Therefore, even titers below the 0.5 EU/mL threshold, which is the official regulation for Abs titers in the EU regarding vaccination Abs thresholds (Regulation No 998/2003, effective 3 July 2004), offer valuable insights into the detection and likely circulation and maintenance of lyssaviruses in bats. This suggests that a large number of bats in the Cat Ba caves had been previously exposed to lyssaviruses and had developed an immune response to them. A high seroconversion value (>4 EU/mL) in one bat sample is noteworthy, potentially indicating a recent, high-titer exposure event or a robust individual immune response.

Four of the five bat species studied, *H. armiger*, *H. alongensis*, *H. poutensis*, and *M. pilosus*, had various levels of *Lyssavirus*-Abs titers. The observed 29% seroprevalence of *Lyssavirus*-specific Abs among cave-dwelling bats on Cat Ba Island provides compelling evidence of the active circulation of various lyssaviruses and prior exposure within these populations. While our observed seroprevalence is noteworthy, the relatively small sample size for particular species limits our ability to generalize these findings broadly. Interpretation of species-specific findings is subject to the inherent limitations of varying sample sizes. Notably, *T. s melanopogon* (n = 2) and *M. pilosus* (n = 6) were represented by very small numbers of individuals, which precluded the robust estimation of their true seroprevalence or seroconversion rates. Therefore, observations for these species must be interpreted with extreme caution and should not be overinterpreted as representative of their broader populations. Future research necessitates larger, targeted sampling of these less frequently captured species to derive more reliable and representative estimates of lyssaviruses exposure and prevalence.

The *Lyssavirus*-glycoprotein exhibits regional sequence variations, a phenomenon commonly observed in its epidemiology. Crucially, *Lyssavirus*-glycoprotein is highly conserved in regions responsible for inducing broad neutralizing Abs. UniProt entries for *Lyssavirus*-glycoprotein (e.g., P16288 for strain SAD B19 or P03524 for strain ERA) characterize it as the primary surface antigen and often detail natural variants. Still, the conserved nature of critical Abs-binding epitopes allows for broad detection. Studies have consistently shown that the commercial PLATELIA RABIES II kit (Bio-Rad Laboratories, Marnes-la-Coquette, France) is effective in detecting Abs against *Lyssavirus* variants from various geographic regions, including Asia, Europe, and North America [41,42,46,47,50]. This broad reactivity strongly suggests that the kit’s antigen generally represents circulating *Lyssavirus*-glycoprotein variants. Although the specific genetic similarity between the antigen in the kit and circulating strains of the genus *Lyssavirus* in Vietnam has not been directly tested, the presence of these conserved glycoprotein regions, which are critical for Abs binding, supports the applicability of the kit. Nevertheless, further validation of the antigen against local strains could strengthen the relevance of the results.

The absence of concurrent nucleic acid testing for direct detection of lyssaviruses limited our ability to confirm active infection or viral shedding at the time of sampling, as well as to assess ongoing transmission within the population. This is primarily due to the nature of lyssavirus infection, characterized by viral migration to target organs and fluctuations in the virus’s circulation over time, which makes direct detection challenging. Therefore, the absence of direct viral detection and broader screening for other lyssaviruses highlights important areas for future research better to understand the *Lyssavirus* genus landscape in cave-dwelling bats.

Historical records mention a possible isolate from lyssaviruses in an Indian bat, a case of human rabies following bat contact [51], and a bat isolate from Thailand [52], both of which are from pteropodid bats. The elevated seroprevalence of lyssaviruses reported in recent studies across several countries in the region, including the Philippines, Cambodia, Bangladesh, and Sri Lanka, provides serological evidence of infection with lyssaviruses in bats, indicating the presence and circulation of these viruses throughout the Indochinese peninsula and highlighting the public health concern of bat-borne zoonotic viruses [7,9,53,54,55]. In the Philippines, 1013 bats were assessed, revealing no evidence of rabies [55], and 478 bats in Malaysia similarly showed no signs [55]. Investigations have also been conducted on lyssaviruses affecting several bat species in North Vietnam [8]; however, they have not focused on the *Lyssavirus* genus serology of cave-dwelling bats inhabiting Cat Ba Island. The current research on anti-*Lyssavirus* Abs levels in the bat community in this area addresses an essential gap in our understanding of zoonotic risk. A thorough study on bat lyssaviruses in Vietnam by [8] found that a significant percentage (24.5%) of the 789 serum samples analyzed exhibited neutralizing Abs against at least one of the tested lyssaviruses. These results confirm that lyssaviruses are not limited to specific geographic areas and are present in bat populations across the region.

### 4.2. Bats as Asymptomatic Reservoirs

Importantly, the obtained serological data came from bats showing no signs of disease, which supports their known status as asymptomatic reservoir hosts [15,18]. The detected Abs in the bats studied on Cat Ba Island could be interpreted as evidence of prior exposure, potential viral clearance, or a controlled infection where the bat does not develop the full-blown disease. This is consistent with the known dynamics and prevalence of the lyssaviruses in bats, where Abs can be detected throughout the progression of clinical disease, which is not necessarily fatal to bats [35,56]. Abs in healthy bats are a fascinating aspect of lyssaviruses biology [4,54,56], as bats can exhibit and maintain Abs against them without succumbing to the fatal disease [56,57]. Unlike in other mammalian species, where Abs against lyssaviruses are only detectable in the final stage of infection, shortly before the animal’s death, seroconversion in repeatedly captured bats can be observed [58]. This finding is consistent with those from other serosurveys in Southeast Asia, such as those in Thailand and the Philippines [52,54]. This phenomenon is a key feature of worldwide bat-borne epidemiology of the lyssaviruses. It enables the virus to persist in bat populations and potentially transmit to other animals or humans. While it is generally accepted that rabies is almost always fatal in humans, carnivores, and laboratory rodents, scientists have no consensus regarding infection with lyssaviruses in bats. For decades, at least two significant issues have been debated: the potential for a “carrier” state, e.g., prolonged virus shedding in saliva without clinical central nervous system infection [59,60,61,62,63], and the issue of non-bite transmission [64,65].

Bats’ unique immune system adaptations allow them to coexist with viruses, such as lyssaviruses, without showing clinical symptoms of disease [21]. A key aspect of their innate immune system is the constitutive expression of type I interferons (IFNs), particularly IFN-α, which is expressed at baseline levels in unstimulated bat tissues and cells. This constitutive IFN expression leads to the continuous activation of interferon-stimulated genes (ISGs), which provide antiviral activity and resistance to DNA damage, creating an environment that limits viral replication while avoiding excessive inflammation. [23,24]. Additionally, the constitutive IFN activity in bats may result in low-level viral shedding, which, combined with their robust innate immune responses, could contribute to the asymptomatic carriage of lyssaviruses [66]. These findings underscore the importance of considering both the innate and adaptive immune responses in bats when interpreting serological data and studying their role in the epidemiology of lyssaviruses. Future research should investigate the interplay between constitutive IFN expression and humoral immunity to better understand the mechanisms underlying viral persistence and control in bats.

The asymptomatic state in bats likely results from a balance between viral replication and the immune response [21], possibly affected by viral glycoprotein levels. Bats may have unique immune strategies or carry viral variants enabling this interaction. The lack of reported bat rabies cases does not mean there is no risk, but rather indicates a silent reservoir that requires surveillance to prevent zoonotic spillover.

Other studies [67,68] suggest that bats infected with lyssaviruses eventually show clinical signs, although the time before these signs appear can vary. Some studies [60,61,62,63] indicate that virus shedding usually happens in the days leading up to clinical symptoms or support the absence of a true “carrier state” in healthy bats. However, bats might shed the virus during early pre-clinical infection stages. They may control infection due to their unique immune systems [59], which can clear the virus before symptoms or limit its replication. For example, in northern Vietnam [8], many healthy bats had neutralizing Abs against a variety of lyssaviruses despite no active virus being present in their brains, indicating prior exposure. Similarly, in Slovakia [69], some healthy bats had *Lyssavirus* Abs, indicating virus circulation without widespread mortality. This ability to carry lyssaviruses without exhibiting symptoms makes bats key reservoirs, reflecting their unique interactions with pathogens.

### 4.3. Analysis of Sex-Related and Species-Specific Differences

The observed sex-related differences in seroprevalence, with a higher detection rate in females overall and a statistically significant difference between sexes in *H. alongensis* and *H. poutensis*, warrant further investigation. These variations may be attributed to sex-specific behavioral patterns, such as differences in roosting aggregation [4], social structures [70], or foraging ranges, which could influence the likelihood of exposure. For example, female bats often form large maternity colonies, which can potentially increase contact rates and transmission opportunities within these densely aggregated groups. Alternatively, physiological factors, including hormonal influences on immune responses, might play a role in differential susceptibility or antibody persistence [71]. Similarly, the elevated seroprevalence in *M. pilosus* compared to other species suggests species-specific ecological or biological factors, such as higher intraspecific contact rates, unique roosting preferences within the cave system, or inherent immunological differences, contributing to varied exposure or infection dynamics within the bat community [72]. These findings underscore the need for detailed ecological and behavioral studies to fully elucidate the complex interplay of factors influencing the epidemiology of lyssaviruses in diverse bat populations.

### 4.4. Ecological Factors and Potential for Lyssavirus Transmission in Cave Environments

The obtained results underscore the crucial importance of cave environments for the transmission of lyssaviruses due to their stable microclimates and protection from predators, making them ideal roosting sites for large bat colonies [19]. These communal roosting habitats foster high bat densities and close interactions [4], which are likely to increase contact rates and the potential for direct virus transmission through bites, scratches, or aerosolized saliva, even in the absence of visible clinical symptoms [73]. Consequently, caves offer superior sites for longitudinal monitoring of bat-virus dynamics due to their stable bat populations and predictable exit times, making cave-dwelling bats a key focus for *Lyssavirus* surveillance.

These cave environments—characterized by dense bat populations and human access points—represent conditions that could facilitate the transmission and evolution of pathogens. Our findings underscore the potential need for thorough ecological surveillance in such environments, not only for known pathogens but also for identifying new viral threats. This highlights the importance of implementing biosecurity measures where human–bat interactions occur. Therefore, targeted surveillance of cave-dwelling bats on Cat Ba Island is crucial for assessing and mitigating potential risks associated with bat-borne lyssaviruses in this ecologically significant region. Ongoing research and proactive public health measures remain essential for addressing the persistent threat of emerging zoonotic diseases from wildlife populations in Vietnam and globally.

## 5. Conclusions

The study detected and quantified IgG anti-rabies viral glycoprotein in 80% (4 out of 5 species) of the cave-dwelling bat species investigated on Cat Ba Island, located north of Vietnam. The species *H. armiger*, *H. alongensis*, *H. poutensis*, and *M. pilosus* exhibited varying antibody titers. The observed 29% seroprevalence of *Lyssavirus*-specific Abs among cave-dwelling bats provides compelling evidence of active lyssaviruses circulation and prior potential exposure within these populations. The presence of viral Abs in healthy bats suggests a capacity for viral tolerance without overt disease. Since cave environments promote close interspecific contact and may facilitate viral transmission, these findings highlight the importance of sustained surveillance in such ecological niches. Continued research and proactive public health interventions are critical to addressing the ongoing threat of emerging zoonotic viruses originating from wildlife in Vietnam and globally.

## Figures and Tables

**Table 1:** *Lyssavirus*-specific anti-glycoprotein antibodies survey of cave-dwelling bats on Cat BA Island, North Vietnam (2024) using the PLATELIA™ RABIES II kit.

Species	Females		Males		Total	
	Npos/Ntotal	Positives (%) (95% CI)	Npos/Ntotal	Positives (%) (95% CI)	Npos/Ntotal	Positives (%) (95% CI)
*H. armiger*	0/18	0% (0–17.59)	4/18	22.22% (9.001–45.21)	4/18	22.22% (9.001–45.21)
*H. alongensis*	4/7	57.14% (25.05–84.18)	6/11	45.45% (21.27–1.99)	10/18	50% (29.03–70.97)
*H. poutensis*	0/6	0% (0–43.53)	1/12	8.33% (1.49–35.39)	1/18	5.55% (0.98–25.76)
*M. pilosus*	1/1	100% (20.66–100)	3/5	60% (23.07–88.24)	4/6	66.66% (30–90.32)

Legend: Npos—number of positive individuals; Ntotal—total number of individuals; CI—confidence interval.

## Data Availability

The original contributions presented in this study are included in the article. Further inquiries can be directed to the corresponding author.
